# Malformation of the Shoulders

**Published:** 1842-06

**Authors:** 


### Selections from American and Foreign Journals.

---

*Malformation of the Shoulders.*—Last week some observations were made on the general condition of Mr. Nellis, the man without arms, with reference to the power acquired by the toes in the performance of motions that would at first seem impossible to be accomplished by any other means than the fingers. Another opportunity has occurred for a re-examination; and it may possibly subserve the interest of some anatomist or physiologist, if we give the following continuation of last week's notice.

In consequence of being destitute of arms, there not being a single vestige of an arm-bone connected with the shoulder-blades, a peculiar control from early infancy has been acquired over the muscles of the mouth, altogether new and surprising. It reminds one of Lamarck's notion of the manner in which animals obtained their forms—from the operation of the force of desire. The elephant, for example, had a short neck, and having a strong desire to clip the tender grass at his feet or the foliage above his head, kept on desiring from age to age, till finally the upper lip was actually elongated into the now characteristic trunk, dragging the nose in its long train. So it would be, were the doctrine true, in regard to the armless descendants of Mr. Nellis, should there be any; the period would arrive when the lips would take the form of a proboscis!

A new labor devolves upon all the facial muscles, and especially upon those about the forehead. This is observable in holding on the hat, throwing it off, and so on. A spinal disease put a stop to Mr. Nellis's growth in his thirteenth year, and he only measures four feet five inches, and weighs but eighty-four pounds. Yet he is quite strong, especially with the teeth and jaws. A fifty-six is easily raised and thrown some feet, by the teeth, which are large, sound, and well set.

Singular as it may appear, in infancy he managed to creep about as well as other children. There is a peculiarity in his vision, worth the special attention of philosophical visiters. It is this: although the most delicate cuttings in paper are executed by the toes, which necessarily removes the work to a considerable distance from the eyes, his vision at the point of the scissors is minutely distinct. This, like the training of the muscles of the lips and feet, was established by the law of necessity;—nature accommodated the organ to the circumstances of the individual. Objects, such as a book, placed at the usual focal distance, soon produce a painful sensation.

One of the anatomical singularities in Mr. Nellis's organization, is that there are no rudimentary arms. The glenoid cavities are, apparently, completely formed, feeling under the finger as though the ball of the humerus were but just slipped aside. The acromion processes jut out boldly, like a protecting roof, on both sides, and on them, as on two projecting hooks, is hung his coat. All the muscles of the scapula are perfectly developed, and he has such perfect control over them too, that he can throw off a coat or re-adjust a garment to its proper place, about as readily as people who have hands.

These few facts in relation to the actual condition of the unfortunate individual to whom they relate, may be of use hereafter to the physiologist. At all events, it would be quite inexcusable not to notice an anomaly so striking. Mr. Nellis says that he cannot imagine any use for a pair of arms, and therefore manifests no regrets that he is without them.—*Boston Med. and Surg. Journ.*

